# Interactions of Mitochondrial Transcription Factor A with DNA Damage: Mechanistic Insights and Functional Implications

**DOI:** 10.3390/genes12081246

**Published:** 2021-08-15

**Authors:** Krystie Chew, Linlin Zhao

**Affiliations:** Department of Chemistry and Environmental Toxicology Graduate Program, University of California, Riverside, CA 92521, USA; kchew003@ucr.edu

**Keywords:** DNA modification, DNA-protein interaction, DNA packing, epigenetics, G-quadruplex, nucleoid, post-translational modification

## Abstract

Mitochondria have a plethora of functions in eukaryotic cells, including cell signaling, programmed cell death, protein cofactor synthesis, and various aspects of metabolism. The organelles carry their own genomic DNA, which encodes transfer and ribosomal RNAs and crucial protein subunits in the oxidative phosphorylation system. Mitochondria are vital for cellular and organismal functions, and alterations of mitochondrial DNA (mtDNA) have been linked to mitochondrial disorders and common human diseases. As such, how the cell maintains the integrity of the mitochondrial genome is an important area of study. Interactions of mitochondrial proteins with mtDNA damage are critically important for repairing, regulating, and signaling mtDNA damage. Mitochondrial transcription factor A (TFAM) is a key player in mtDNA transcription, packaging, and maintenance. Due to the extensive contact of TFAM with mtDNA, it is likely to encounter many types of mtDNA damage and secondary structures. This review summarizes recent research on the interaction of human TFAM with different forms of non-canonical DNA structures and discusses the implications on mtDNA repair and packaging.

## 1. Introduction

Mitochondria are important organelles in eukaryotic cells due to their wide variety of functions in energy production, cell signaling, and biosynthesis [1,2,3]. Mitochondria possess genetic material, mitochondrial DNA (mtDNA), which is a circular, double-stranded molecule with 16,569 base pairs and multiple copies [3,4]. Even though each mtDNA molecule is much smaller than nuclear DNA in size, mtDNA encodes two ribosomal RNAs, 22 transfer RNAs, and 13 protein subunits of the oxidative phosphorylation system. Therefore, the integrity of mtDNA is critically important for cellular and organismal functions [3,4]. Compromised mtDNA, in the form of point mutations, deletions, and depletion, has been associated with a variety of mitochondrial disorders and other diseases such as diabetes, neurodegeneration, and cancer [4,5,6,7].

mtDNA is more susceptible to chemical and physical factors compared to its nuclear counterpart [4,8]. Nonetheless, mitochondria possess unique pathways to maintain the integrity of mtDNA, such as degradation of damaged DNA molecules and mitochondrial content exchange through fission and fusion [3,4,9,10]. In addition, mitochondria can repair damaged DNA molecules via base excision repair (BER), along with several other less well-defined pathways [11,12]. Notably, mitochondria appear to lack nucleotide excision repair (NER) activities [11,12], which is crucial for alleviating deleterious effects from bulky DNA modifications in the nucleus. 

mtDNA is organized into DNA-protein complexes known as nucleoids. The nucleoids are located in the matrix between the cristae tubules and are separated from the inner boundary membrane by cristae. Each nucleoid typically contains a single copy of mtDNA [13,14]. Nucleoid proteins consist of a list of approximately forty proteins, including key enzymes in DNA and RNA metabolism and other DNA-binding proteins [9,15,16,17]. Mitochondrial transcription factor A (TFAM) is one of the two most abundant nucleoid proteins, with the other being mitochondrial single-stranded DNA-binding protein (mtSSB) [15,18]. TFAM is the primary protein that packages mtDNA into the condensed nucleoid structure. Because nucleoid proteins interact extensively with mtDNA, it is inevitable that they encounter various DNA lesions. TFAM, for example, has been noted to bind to certain types of DNA lesions and potentially affect their repair processes [19]. As such, interactions between TFAM and damaged mtDNA are critical for the initiation and regulation of mtDNA repair. This review summarizes recent advances in the biochemical basis of human TFAM-DNA interactions with a focus on the interaction of TFAM with different forms of non-canonical DNA structures. For general discussions on the structure, function, and physiological importance of TFAM, please see excellent reviews [20,21,22,23,24]. 

## 2. Structures of TFAM-DNA Complexes

**Figure 1:**
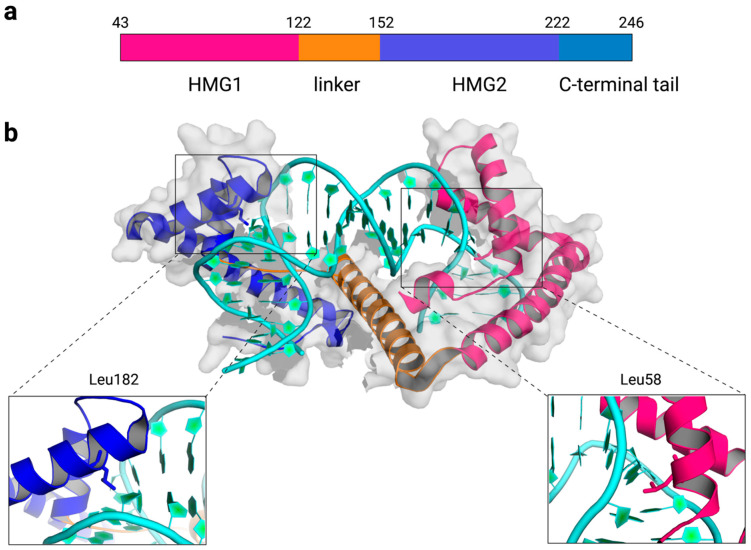
Overview of the TFAM-DNA complex. (**a**) The domain structure of the mature form of human TFAM without the mitochondrial targeting sequence (amino acid 43–246). (**b**) The crystal structure of human TFAM-DNA containing the light strand promoter (LSP) sequence (PBD: 3TQ6). Key DNA-intercalating residues, L58 and L182, are highlighted in sticks.

TFAM is a high-mobility group B protein that is made of two high-mobility group (HMG) box domains, HMG1 and HMG2, separated by a linker region, and a C-terminal tail (Figure 1a). Each HMG-box domain is composed of three helices that are arranged into an L-shape. The concave surface of each HMG-box domain intercalates amino acid residues into the DNA minor groove to distort DNA into a U-turn structure [25,26,27,28]. DNA bending is stabilized by the interdomain linker, which compensates for the repulsion between backbone phosphates of DNA that are brought closer by the DNA U-turn, by several polar and nonpolar interactions, and by the intercalation of nonpolar residues from helices 1 and 2. Various amino acids are inserted between the DNA bases to induce DNA bending, but a maximal distortion of the DNA double helix is observed at nucleobases near DNA-intercalating residues, L58 of HMG1 and L182 of HMG2 (Figure 1b). TFAM can bind to and induce a U-turn structure with both specific and nonspecific DNA sequences [25,26,27,28]. According to the crystal structures of TFAM with the light strand promoter (LSP) in mtDNA [25,26], the heavy strand promoter 1 (HSP1) [27], and a nonspecific DNA sequence derived from the *ATPase6* gene [27], TFAM bends these DNA sequences and results in similar structures overall. Results from Förster resonance energy transfer (FRET) assays corroborate the U-turn structures observed in static crystal structures, reinforcing the U-turn shape of TFAM-DNA complexes in a solution environment [28,29]. 

Despite the structural similarities observed in the aforementioned crystal structures, additional crystallographic studies of TFAM-DNA complexes have revealed variations in the U-turn shape. Using additional TFAM-binding sites at the control region of mtDNA (termed Site-X and Site-Y), Cuppari et al. solved crystal structures of TFAM-DNA complexes and compared the structures with those with LSP and nonspecific sequences [28]. Overall, the two HMG-box domains retain similar structures; however, the linker region shows considerable differences. When three crystal structures with LSP, Site-X, or Site-Y were superimposed at HMG1 or HMG2, a slight progressive distortion was observed at the linker region, which results in reorientations of the other HMG-box domain. TFAM-DNA complexes show a stiffer conformation when TFAM is bound to Site X and a more flexible conformation when TFAM is bound to Site Y. Such difference in TFAM-DNA conformations is modulated by sequence-dependent properties, as indicated in MD simulations with free LSP, Site-X, and Site-Y sequences. Together, these results suggest that TFAM binds mtDNA in a non-uniform manner and potentially results in an uneven distribution of aggregation sites. Such a model is consistent with the nucleoid formation process initiated by TFAM aggregation [30,31] and cross-strand binding observed in vitro [13] and in living cells [32], and an uneven distribution of TFAM on mtDNA in cell cultures based on DNase-seq analysis [33]. 

## 3. Dynamics of TFAM-DNA Interactions

TFAM-DNA complexes are dynamic in that (i) the two HMG domains undergo butterfly or ‘‘breathing’’ movement [29,34] and (ii) the linker region can unfold reversibly, as demonstrated by MD simulations [29]. The dynamic equilibrium is governed by protein-DNA interactions, the unfolding and refolding of the linker region, and the intrinsic tendency of DNA to assume a straighter conformation. The linker region in free TFAM is disordered but gains an α-helix structure upon binding to DNA [29,35]. While DNA will tend toward its free conformation, this inclination is counteracted by the folding and unfolding of the linker, which restores the U-turn structure in DNA [29]. In addition, Small-angle X-ray scattering (SAXS) experiments suggest that TFAM-DNA complexes are heterogeneous owing to the intrinsic flexibility of the C-terminal tail [29], modulation by DNA sequence [28], and binding of TFAM with different DNA sequences in multiple orientations [28]. 

TFAM binds and kinks DNA in a stepwise manner, as revealed in FRET assays [29]. First, HMG1 binds and bends DNA into a V-shape. This reduces the accessible conformational space for the linker to bind, so it folds into an α helix structure and wraps around the DNA, subsequently reducing the distance between HMG1 and HMG2. Then, HMG2 associates with DNA, which induces a second V-kink to form the complete U-turn structure. The binding of TFAM increases the intrinsic flexibility of DNA by locally denaturing DNA and forming thermal openings in double-stranded DNA [30,36]. If the small bubbles induced by TFAM binding are less than ten base pairs apart from each other, they will interact and merge into a larger bubble that acts as a flexible hinge in the DNA molecule [36]. The flexible hinge can occupy a range of angles around a slightly preferred bending angle of 180° [30,34]. 

Similar to many other DNA-binding proteins, TFAM diffuses extensively over DNA and forms patches of TFAM in a cooperative manner, as evidenced by observations in vitro [13,30,31] and in living cells [32]. According to single-molecule studies, TFAM binds more stably next to an already bound TFAM compared to bare DNA [30,31]. This may result from the diffusing TFAM sensing structural changes in DNA induced by already bound TFAM proteins. The local destabilizing effect may promote the binding of TFAM to DNA upon its collision to DNA-bound TFAM, resulting in protein aggregation [30]. As such, changes in the degree of flexibility induced by local melting can regulate DNA compaction. Both HMG1 and HMG2, together with the linker region, facilitate DNA compaction, as demonstrated by tethered particle motion (TPM) assays [27]. In addition, the mobility of the protein can affect the effectiveness of the protein-induced bubble and, subsequently, the flexibility of DNA [34]. If TFAM is slower or less mobile, it will be able to form a larger and more stable hinge more effectively. Larger hinges have been proposed to contribute more to DNA compaction compared to smaller hinges that contribute more to specific binding required for transcription initiation [36]. Both TFAM sliding and DNA melting are thought to be necessary for effective and specific transcription regulation by TFAM. TFAM sliding may facilitate localization to a specific binding site in the promoter region because TFAM does not immediately stably bind to DNA upon contact [30]. Collectively, these data demonstrate the dynamic characteristics of TFAM and TFAM-DNA complexes and underscore the importance of considering these properties when interpreting data from equilibrium conditions or from living cells. 

## 4. Binding of TFAM to Non-Canonical DNA Structures

### 4.1. Oxidative Damage

**Figure 2:**
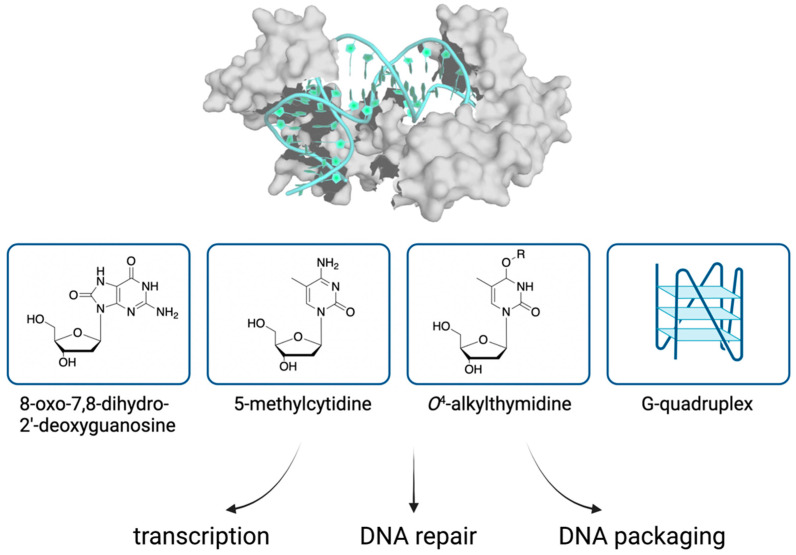
Binding of TFAM to DNA modifications and implications on the organization and transactions of mitochondrial DNA. TFAM (PDB: 3TQ6) and DNA are shown in surface and carton views, respectively.

TFAM is crucial for packaging DNA and extensively interacts with the mtDNA genome. As such, it is likely to encounter various kinds of DNA lesions. 8-oxo-7,8-dihydro-2′-deoxyguanosine (8-oxodG, Figure 2), an oxidized derivative of deoxyguanosine, is one of the most abundant DNA modifications formed by exposure to reactive oxygen species [37]. 8-oxodG is a marker of oxidative DNA damage and is present in mtDNA. Despite the generally perceived highly oxidative environment in mitochondria, the relative levels of mitochondrial and nuclear 8-oxodG remain controversial due to the different biological samples, detection methods [38,39,40,41,42,43], and the potential for the artificial formation of 8-oxo-dG during sample workup [44]. TFAM has been shown to bind more efficiently to a 22-nt double-stranded (ds) oligodeoxynucleotide containing an 8-oxodG relative to an unmodified substrate using electrophoretic mobility shift assays (EMSA) [45]. Interestingly, TFAM also binds preferentially to substrates A:8-oxodG or C:8-oxodG pairs compared to those bearing mispairs with T or G. Furthermore, between the HMG1 and HMG2 domains, HMG1 binds stronger to substrates with 8-oxodG, whereas HMG2 has no detectable binding activities. The results are consistent with the stronger DNA-binding activity of the HMG1 domain when comparing several truncated TFAM variants [35]. These data suggest that HMG1 may play a more important role in recognizing altered DNA compared to HMG2 [45]. On the other hand, when longer dsDNA substrates (91 nt) were used, TFAM shows a modest preference for the 8-oxodG-containing substrate over an unmodified substrate [19], suggesting that the preferential binding depends on the sequence and length of oligodeoxynucleotides. Additional studies are required to clarify the structural and molecular mechanisms of the observed binding of TFAM to 8-oxodG-containing substrates. 

### 4.2. DNA Methylation

5-Methylcytidine (5mC) is a common form of epigenetic modification that functions in gene regulation (Figure 2). Historically, the existence of the reversible methylation of mtDNA has been a topic of debate (reviewed in [46,47,48]). However, with the advancement of DNA sequencing techniques, accumulating evidence supports the presence of such modifications in mtDNA [49,50,51]. Emerging research continues to reveal the regulatory and pathological roles of mitochondrial epigenetic modifications [52]. CpG sequences are regions of DNA where cytosine is followed by guanine in the 5′ to 3′ direction. Low levels of 5mC at CpG sequences (5mCpG) have been detected in mitochondrial DNA [49], but the role of 5mCpG in mitochondria remains unclear. Recently, Dostal and Churchill studied the effect of 5mCpG on TFAM-DNA binding using biochemical assays [53]. Compared to nonmethylated DNA substrates, 5mCpG in the HSP1 increases the binding affinity of TFAM and induces TFAM multimerization. Such effects were not observed with nonspecific DNA sequences. In vitro transcription assays revealed a stimulatory effect on transcription when 5mCpG is in HSP1 and HSP2 substrates. The extent of stimulation relative to controls depends on the concentration of TFAM. By contrast, a modest decrease in transcription activities was observed with the LSP sequence. The different effects with different DNA substrates highlight the importance of DNA sequence on TFAM-DNA binding. Methylation of cytosine is not associated with significant changes to B-DNA structure, but DNA methylation can increase the rigidity of DNA through stabilizing base stacking. As the authors proposed, it is likely that cytosine methylation impacts the relative stability of the TFAM-DNA complex by altering the stacking and deformability of DNA in HSP1. Besides, the 5mCpG in HSP1 is adjacent to the intercalation sites and is likely to make further contacts with the C-terminal tail of TFAM. This allows for further interactions that increase base stacking stability and can contribute to the increased affinity of TFAM for 5mCpG DNA. 

By contrast, the presence of a different DNA methylation modification, *N*^6^-methyldeoxyadenosine (6mdA), attenuates TFAM-DNA binding [54]. 6mdA is an emerging regulator in eukaryotic nuclear and mitochondrial genomes. Hao et al. showed that the presence of 6mdA in a HSP1-based probe compromised DNA binding, as evidenced in pull-down assays and EMSA [54]. Interestingly, such an effect was not observed with an LSP probe. In addition, the presence of 6mdA decreases the overall yield of products in in vitro transcription assays. Further, the authors observed the attenuation of DNA bending by TFAM in the presence of 6mdA with HSP and LSP substrates. Future research is required to clarify the role of 6mdA in mtDNA packaging. Overall, the results are consistent with the crucial role of DNA sequence in modulating TFAM-DNA binding (*vide supra*) [28]. Considering the recent data demonstrating that mtDNA is methylated predominantly at non-CpG contexts [49], additional research on the methylation at non-CpG regions is warranted for a complete understanding of the functional impact of DNA methylation on TFAM-DNA interactions. 

### 4.3. O^4^-Alkylthymidine DNA Lesions

Alkylated DNA lesions are a class of DNA damage products that results from exposure to environmental toxicants and/or endogenous metabolites. Alkylating agents can react with nucleobases and the phosphate backbone to form DNA lesions that can block DNA replication and transcription and induce DNA mutations [55,56]. Thymidine can be alkylated to generate *O*^2^-alkyldT, *O*^4^-alkyldT (Figure 2), and *N*3-alkyldT [56]. While *N*3-alkyldT can be efficiently repaired, *O*^2^-alkyldT and *O*^4^-alkyldT lesions are poorly repaired and tend to persist in mammalian tissues [57,58]. The formation *O*^2^-pyridyloxobutylthymidine in mtDNA has been demonstrated using samples from rats treated with tobacco-specific *N*-nitrosamines [59]. To better understand the repair of *O*^2^-alkyldT and *O*^4^-alkyldT lesions, He et al. identified several candidate proteins using alkylated thymidine lesion-containing DNA probes and quantitative proteomics [60]. TFAM is among the list of proteins that bind *O*^4^-alkyldT lesions. The binding capability of TFAM to *O*^4^-alkyldT lesions, *O*^4^-nButylthymidine, and *O*^4^-pyridyloxobutylthymidine was confirmed using EMSA. Compared to lesion-free DNA, TFAM prefers to bind to both types of *O*^4^-alkyldT lesion-containing DNA. Given that HMG1 of TFAM plays a predominant role in DNA binding, the binding affinity of each domain alone was also compared. HMG1 showed higher binding selectivity for *O*^4^-alkyldT DNA compared to full-length TFAM, while HMG2 displayed weak binding to both lesion-free and lesion-containing DNA. As such, the authors concluded that the preferential binding of *O*^4^-alkyldT DNA by TFAM is largely due to the HMG1 domain. TFAM binding to these lesions was also found to enhance transcriptional bypass [60]. While TFAM overexpression did not alter the efficiency of transcriptional bypass, it did elevate the amount of transcriptional mutagenesis of the *O*^4^-alkyldT lesions through promoting the misincorporation of guanosine across the lesion site during transcription. However, the mechanism through which TFAM binding modulates transcriptional bypass remains unknown. Additional research is warranted to clarify the mechanism and biological significance of the observed binding. 

### 4.4. Abasic Sites

Abasic (AP) sites are the most abundant type of endogenous DNA damage in cells [37,61]. AP sites are cytotoxic and mutagenic, with the potential to form additional secondary DNA lesions due to their chemical reactivity, which together contribute to their adverse effects [62]. Unlike oxidative DNA lesions, TFAM shows no significant binding preference for DNA substrates containing a tetrahydrofuran (AP analog) modification under equilibrium binding conditions [63] and using EMSA [19,63]. However, TFAM has been shown to cleave AP-DNA to form strand cleavage in vitro and in cellular extracts and is proposed to play a role in damaged mtDNA degradation through facilitating DNA turnover [63]. mtDNA degradation is a pathway that is activated when mitochondria are unable to repair the damage, or if the amount of damage has exceeded the repair capacity [64]. It is nonspecific to lesion type as it occurs with multiple DNA lesions, including abasic sites. mtDNA degradation has been shown to be an essential player in maintaining the integrity of mitochondrial genomes [9]. Given the abundance of TFAM in mitochondrial nucleoids, it is conceivable that TFAM can play such a role in modulating the stability of AP-DNA. Notably, the TFAM-mediated AP-DNA cleavage produces TFAM-DNA cross-links as reaction intermediates [63], which raises the possibility that the reaction may increase the resident time of TFAM on AP-containing DNA. Future research is required to confirm the participation of TFAM in such reactions and the role of TFAM in DNA degradation *in cellulo* and in vivo. 

### 4.5. Other Non-Canonical DNA Structures

G-quadruplexes (G4s) are four-stranded, non-canonical secondary structures that tend to form in DNA sequences rich in guanine [65]. G4s play an emerging role in a myriad of biological functions, including transcription, replication, genome stability, epigenetic regulation, and cancer biology [65,66,67,68]. The existence of G4s has been confirmed in mtDNA of live cells using small molecule G4 ligands [69]. G4s have been shown to perturb mtDNA replication, transcription, and respiratory function in non-cancerous cells [70]. In vitro, a specific G-quadruplex-forming sequence at HSP1 blocks DNA synthesis by DNA polymerase γ [71]. Additional effects of mitochondrial G4 are summarized in a recent review [72]. TFAM has been shown to bind DNA or RNA G4s with high affinity in vitro [73]. Given that TFAM coats mtDNA, it is possible that its interactions with G4s can regulate mtDNA organization. A pattern of 29 mtDNA genomic footprinting (mt-DGF) sites were found to be shared between samples regardless of tissue or developmental stages [33]. These common sites colocalize with known mtDNA regulatory elements, including G4 structures; however, the mt-DGF pattern correlates with TFAM-poor sites [33]. These data suggest that TFAM may not or only bind transiently to G4 structures in vivo. The discrepancy underscores the importance of verifying the binding of TFAM to G4s and demystifying the biological functions of G4s in mitochondria in vivo. 

## 5. Post-Translation Modifications of TFAM

The post-translation modification (PTM) of TFAM could be an important mechanism for altering its DNA-binding activity, protein-protein interactions, homodimerization or cooperative binding characteristics to regulate transcription initiation and mtDNA compaction [20]. Several studies have demonstrated the effect of PTM of TFAM using phosphoserine (D substitution) and acetyl-lysine (Q substitution) mimics. For example, TFAM phosphorylation at S55, S56, S61 in HMG1 and S160 in HMG2 have been detected in HEK293 cells overexpressing TFAM [74]. To examine the effect of TFAM phosphorylation in cells, several phosphoserine mimicking variants are expressed in human HeLa cells. Because mitochondrial Lon protease is an important regulator of TFAM level and is known to degrade DNA-free TFAM, the sensitivity of the TFAM level to Lon was used to infer the DNA-binding capability of TFAM phosphoserine mimics [74]. 

It has been shown that variants containing phosphoserine mimics in HMG1, but not HMG2, can be degraded by Lon [74], suggesting that phosphorylation of TFAM impairs DNA binding. The results are consistent with the involvement of S55 and S56 in interacting with DNA in crystallographic studies [25,26]. Similarly, TFAM phosphomimics at S55 and S56 exhibit reduced activity in transcription assays. Phosphorylation likely causes electrostatic repulsion of the DNA phosphate backbone, thereby affecting DNA binding and allowing TFAM to be degraded by Lon. This is potentially an important mechanism for DNA decompaction as phosphorylation leads to the release of TFAM from mtDNA. Subsequent degradation by Lon allows for sustained DNA decompaction. As such, the coordination of phosphorylation and dephosphorylation with proteolysis of TFAM may be a mechanism by which TFAM and mtDNA are maintained [74].

In human neuroblastoma (SH-SY5Y) cells treated with complex I inhibitor 1-methyl-4-phenylpyridinium (MPP^+^), TFAM is phosphorylated in an extracellular signal-related protein kinase (ERK)-dependent manner [75]. Under these conditions, ERKs are activated and phosphorylate TFAM to suppress mitochondrial biogenesis. Phosphorylation occurs at S177 as shown by mass spectrometric analysis. The phosphomimic variant (TFAM S177D) showed reduced binding to LSP, but not to nonspecific DNA, as shown in DNA-specific pull-down assays using MPP^+^-treated cells [75]. In addition, the phosphomimic variant fails to restore the level of transcription products when it is expressed under endogenous TFAM knockdown conditions. Moreover, the S177D variant is unable to support mitochondrial respiration. 

Lysine acetylation is a prevalent modification in mitochondria [76]. Indeed, a number of lysine residues (K62, K76, K111, and K118) of TFAM have been shown to be acetylated in human HEK293 cells [77]. To compare the effect of acetylation and phosphorylation of TFAM on TFAM-DNA interactions, acetyl-lysine (Q) and phosphoserine (D) mimics of human TFAM have been studied using ensemble and single-molecule methods [77]. Acetyl-lysine and phosphoserine mimics of human TFAM have been shown to compact nonspecific DNA, but only when the mimics were in high concentrations, indicating that both mimics exhibit reduced DNA-binding affinities. The reduced binding affinity of the acetyl-lysine mimic is attributed to a decreased on-rate of DNA-binding, whereas the phosphoserine mimic exhibits a decreased on-rate and an increased off-rate. The addition of an acetyl group on lysine neutralizes the positive charge and increases steric hindrance that disrupts interactions with the DNA backbone. Phosphorylating serine also introduces steric hindrance as well as a negative charge that leads to electrostatic repulsion with the DNA backbone. Both acetylation and phosphorylation can interfere with TFAM-DNA interactions and affect TFAM activity on mtDNA, albeit via different mechanisms [77].

Although amino acid substitution is commonly used to mimic PTMs, a number of limitations have been discussed previously [78]. For example, a mimicry of a phosphoserine using D substitutes a dianionic phosphate monoesters with a singly charged carboxylate. From a steric perspective, a D is smaller than a phosphoserine. Consequently, D mimics often fail to recapitulate the characteristics of a phosphoserine [78]. Similarly, mimicking lysine acetylation with Q is also known to lead to a less active enzyme under certain conditions [79]. To investigate the effect of lysine acetylation of TFAM, Fang et al. modified recombinant human TFAM using acetyl-CoA-mediated chemical acetylation and identified acetylation sites at K52, K62, K76, K111, and K118 in HMG1 and K126 in HMG2 [80]. The authors used the acetylated TFAM containing the aforementioned modifications to examine the impact of acetylation on the DNA unwinding and binding activities of TFAM. The unwinding activity can be reduced by TFAM acetylation without affecting the DNA-binding ability of TFAM. To compact DNA, TFAM induces negative supercoils where it binds DNA and positive supercoils outside the bound region. Eukaryotic topoisomerase I can relax the positive supercoils so that TFAM can continue to effectively introduce topological modifications to DNA. However, at high concentrations of TFAM, there is a decrease in the supercoiled form. As such, it appears that TFAM can unwind DNA in a TFAM/DNA-ratio-dependent manner. It is possible that at high TFAM concentrations, excess TFAM may inhibit topoisomerase I access to mtDNA, preventing it from relaxing TFAM-induced DNA contortions and rendering TFAM unable to continue supercoiling DNA. Acetylation at lysine residues may ease this inhibitory effect potentially by reducing the cooperativity of TFAM to prevent aggregation and allow access to mtDNA by topoisomerase I. It is likely that not all these sites are modified concurrently in vivo and that the number of residues modified at a time can dictate the biological effect. Because TFAM interacts with DNA through many contacts, modifying a few of the lysine residues is unlikely to completely impair binding. As such, acetylation of TFAM may be an important means to change overall and/or local topology without significantly affecting the stability of mtDNA [80]. 

Overall, these data support the importance of PTM in regulating TFAM-DNA binding and cognate functions. Additional research with TFAM containing canonical PTMs is needed to understand their functions without bias. Considering that multiple types of PTMs may co-exist in a biological context, developing techniques to prepare proteins with multiple PTMs is needed to decipher the function of these proteins. 

## 6. Conclusions and Future Perspectives

It remains challenging to demonstrate the interaction of TFAM with various DNA modifications in cellulo or in vivo partly due to the difficulty of importing modified DNA (or DNA) to mitochondria in cells. In a study to examine the levels of mtDNA damage and TFAM binding in aged rat tissues in vivo, Chimienti et al. used a qPCR-based approach to quantify mtDNA damage [81]. It was shown that the regions encompassing the origins of mtDNA replication (D-loop and Ori-L) contain more DNA-replication blocking lesions (e.g., strand breaks) than a control gene region (ND1). Notably, TFAM has been shown to bind preferentially to these regions using mtDNA immunoprecipitation and qPCR. In light of the evidence for TFAM-binding to 8-oxodG-containing DNA in vitro, the authors also compared the levels of mtDNA damage before and after treating with formamidopyrimidine DNA glycosylase (FPG) and found that aged tissues contain more FPG-sensitive sites with three regions (D-loop, Ori-L, and ND1) following the same trend. Interestingly, another study from the same laboratory noted that the absence of such correlation in extremely aged (32-month-old) rat tissues, implying a role of TFAM-DNA binding in longevity [82]. Nonetheless, the type of mtDNA damage remains unclear, considering that FPG has both DNA *N*-glycosylase and AP lyase activities and can recognize lesions other than 8-oxodG [83,84]. Therefore, caution should be exercised when trying to correlate preferential localization of TFAM at certain regions of mtDNA with potential binding to specific lesions.

The interactions of TFAM may suggest a role of TFAM in regulating DNA repair [19]. For example, the interaction of TFAM with 8-oxodG-containing DNA substrates has been shown to inhibit human 8-oxoguanine DNA glycosylase 1 (OGG1) [19]. Similar effects were also observed for uracil-DNA glycosylase (UDG), AP endonuclease 1 (APE1), and DNA polymerase γ, indicating that the inhibition is not lesion specific. The inhibitory effect is likely due to competition for DNA substrates, as evidenced by the lower inhibitory effect of the TFAM variant (L58A), which is deficient in DNA binding, relative to wild-type TFAM [19]. TFAM is likely to compete with other repair proteins in vivo, given the abundance of TFAM in the mitochondrial nucleoid [18]. Nonetheless, such effects remain to be established firmly in vitro and in vivo. As discussed, the interaction of TFAM with DNA is dynamic, so it is necessary to consider the motions within TFAM-DNA complexes and that of TFAM molecules on mtDNA. In addition, the sequence, length, and topology of DNA could affect how TFAM recognizes and binds DNA and subsequently modulate the effects on repair processes. 

TFAM binding to different non-canonical DNA structures may affect mtDNA packaging. Studies have found that nucleoid formation is a multistep process initiated by protein aggregation and cross-strand binding by TFAM [13,30,31]. Because TFAM binding is cooperative, the protein favorably binds proximally to already-bound TFAM resulting in TFAM clusters on mtDNA [30]. As such, mtDNA packaging could be modulated by interactions with DNA modifications and secondary structures. Recently, phase separation, an emerging organizational mechanism of non-membrane bound cellular structures [85], has been shown to contribute to the organization of mitochondrial nucleoids [32]. TFAM and mtDNA combine and phase separate into viscoelastic, multiphase droplets. This occurs through many weak interactions along the flexible backbone of TFAM. In these droplets, mtDNA is not uniformly distributed but de-mixes from TFAM within the droplet, accounting for its multiphase behavior. To what extent the interaction of TFAM with various non-canonical DNA structures may contribute to phase separation remains enigmatic. 

In summary, the interactions of TFAM with DNA modifications remain an intriguing research area. It remains important to confirm the interactions in living cells and in vivo. Moreover, mechanistic details of the observed interactions have yet to be deciphered. For example, because TFAM-DNA interaction is affected by intrinsic characteristics of DNA, and modified DNA often has non-native conformations, the relative contribution from DNA modifications or DNA conformation to TFAM-DNA binding needs to be clarified. While this review focuses on TFAM, information on how other DNA-interacting proteins contribute to regulating DNA damage and repair is equally important for a complete understanding of mitochondrial genome maintenance. 

## Data Availability

Not applicable.
